# Synthesis of gallium nitride nanostructures by nitridation of electrochemically deposited gallium oxide on silicon substrate

**DOI:** 10.1186/1556-276X-9-685

**Published:** 2014-12-18

**Authors:** Norizzawati Mohd Ghazali, Kanji Yasui, Abdul Manaf Hashim

**Affiliations:** 1 Malaysia-Japan International Institute of Technology, Universiti Teknologi Malaysia, Jalan Semarak, 54100 Kuala Lumpur, Malaysia; 2 Department of Electrical Engineering, Nagaoka University of Technology, Kamitomioka-machi, Nagaoka, Niigata 940-2137, Japan

**Keywords:** Electrochemical deposition, Gallium oxide, Gallium nitride, Nanostructure, Nitridation

## Abstract

Gallium nitride (GaN) nanostructures were successfully synthesized by the nitridation of the electrochemically deposited gallium oxide (Ga_2_O_3_) through the utilization of a so-called ammoniating process. Ga_2_O_3_ nanostructures were firstly deposited on Si substrate by a simple two-terminal electrochemical technique at a constant current density of 0.15 A/cm^2^ using a mixture of Ga_2_O_3_, HCl, NH_4_OH and H_2_O for 2 h. Then, the deposited Ga_2_O_3_ sample was ammoniated in a horizontal quartz tube single zone furnace at various ammoniating times and temperatures. The complete nitridation of Ga_2_O_3_ nanostructures at temperatures of 850°C and below was not observed even the ammoniating time was kept up to 45 min. After the ammoniating process at temperature of 900°C for 15 min, several prominent diffraction peaks correspond to hexagonal GaN (h-GaN) planes were detected, while no diffraction peak of Ga_2_O_3_ structure was detected, suggesting a complete transformation of Ga_2_O_3_ to GaN. Thus, temperature seems to be a key parameter in a nitridation process where the deoxidization rate of Ga_2_O_3_ to generate gaseous Ga_2_O increase with temperature. The growth mechanism for the transformation of Ga_2_O_3_ to GaN was proposed and discussed. It was found that a complete transformation can not be realized without a complete deoxidization of Ga_2_O_3_. A significant change of morphological structures takes place after a complete transformation of Ga_2_O_3_ to GaN where the original nanorod structures of Ga_2_O_3_ diminish, and a new nanowire-like GaN structures appear. These results show that the presented method seems to be promising in producing high-quality h-GaN nanostructures on Si.

## Background

Gallium nitride (GaN) is a very hard, chemically and mechanically stable wide bandgap (3.4 eV) semiconductor material with high heat capacity and thermal conductivity which makes it suitable to be used for sensors [1-8], high power electronic devices such as field-effect transistor (FET) [9] and optoelectronic devices such as light-emitting diode (LED) [10]. Up to this date, many techniques have been explored to synthesize GaN nanostructures including nanowires, nanorods, nanodots and so forth since such low-dimensional nanostructures are promising for increasing the performance optoelectronic devices and the sensitivity of sensors [11,12]*.* For example, GaN nanorods and nanowires have been applied for chemical sensing application as reported by Wright et al. [13] and Huang Y et al. [14], respectively, due to a large surface to volume ratio. GaN nanodots have been used in photodetectors as reported by Kumar et al*.*[15].

Recently, GaN on silicon carbide (SiC) or sapphire substrate have been widely used for several specific electronic applications. However, these substrates are expensive and not available in large wafer size [16]. According to Kukushkin et al*.*, Si substrate seems to be more preferable for the heterostructure growth of GaN due to the availability of Si in large wafer size, the low price of Si and the maturity of Si-based technology [17]. In addition, the integration of GaN-based devices on Si platform seems to be very attractive for the hybrid integration towards ‘More than Moore’ technology [18]. Several vapor-phase techniques have been reported for growing GaN nanostructures directly on Si with high quality which include molecular beam epitaxy (MBE) [19], metal-organic chemical vapor deposition (MOCVD) [20] and hydride vapor phase epitaxy (HVPE) [21]. However, these vapor-phase techniques are too expensive and their growth parameters are quite complicated. In recent years, a transformation of the grown gallium oxide (Ga_2_O_3_) structures on Si to GaN by a so-called nitridation seems to be a simple method to create a GaN/Si heterostructure [22]. Here, a nitridation is achieved by annealing the Ga_2_O_3_ structures in ammonia gas. Li et al. reported the repeatable transformation of the CVD-grown GaN structures to Ga_2_O_3_ structures by an annealing in air and back to GaN structures by an annealing in ammonia [23]. Moreover, there are several studies reporting the formation of GaN nanostructures by annealing the sputtered Ga_2_O_3_ layer on metal-coated Si substrates in ammonia [24-27]. To our knowledge, the nitridation of the electrochemically deposited Ga_2_O_3_ structures on bare Si substrates to form GaN nanostructures without the assistance of metal catalyzers does not appear in the published literature.

Recently, we report the growth of Ga_2_O_3_ nanostructures directly on Si without any assistance of metal catalyzer by using a simple electrochemical deposition [28]. This liquid-phase technique provides several advantages such as high controllability of thickness and morphologies of Ga_2_O_3_ nanostructures due to a less number of growth parameters. In this work, we investigate the formation of GaN nanostructures by ammoniating the electrochemically deposited β-Ga_2_O_3_ nanostructures on Si substrate. Up to this date, no such similar work is reported where a combination of liquid-phase and vapor-phase methods is utilized to form a GaN/Si heterostructure. The effects of the ammoniating times and temperatures were studied. The mechanism for the growth of GaN was proposed and discussed.

## Methods

**Figure 1:**
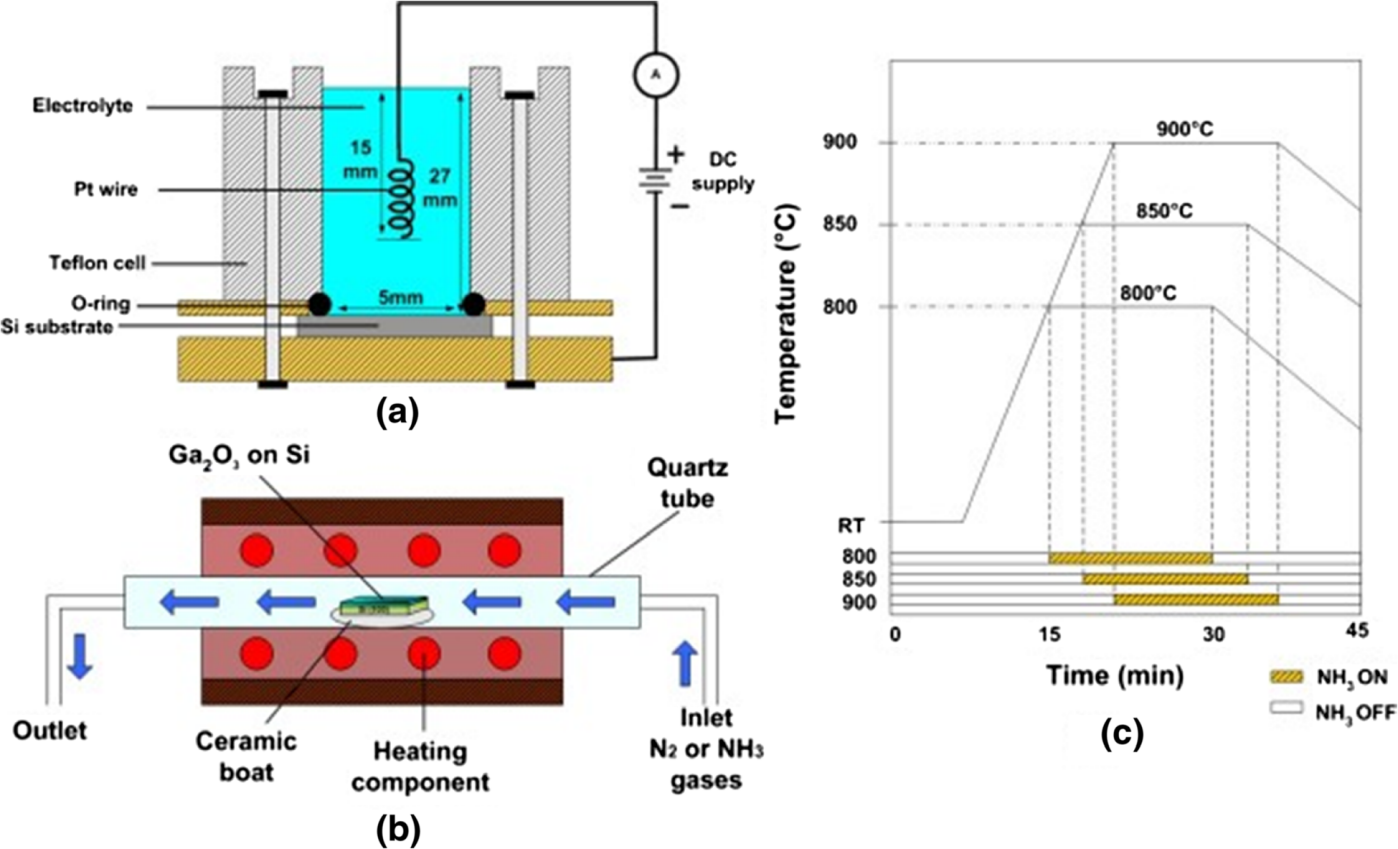
Schematic of (a) electrochemical setup, (b) nitridation set up and (c) growth timing chart.

In this study, the synthesis can be divided into two major processes; i) a formation of Ga_2_O_3_ structures as the ‘seed’ structures and ii) an ammoniating process to transform Ga_2_O_3_ to be GaN. The deposition of Ga_2_O_3_ on Si (100) substrate (resistivity of 15 to 25 Ω · cm) was carried out by a simple two-terminal electrochemical process. In the electrochemical process, a mixture of Ga_2_O_3_ (99.99%), HCl (36%), NH_4_OH (25%) and DI water was used as an electrolyte [28]. Since Ga_2_O_3_ is insoluble in water, HCl is added to dissolve Ga_2_O_3_. The preparation of electrolyte was done as follows. First, Ga_2_O_3_ was dissolved in 1.5 ml HCl. Then, 6.5 ml DI water was added into the solution, followed by NH_4_OH as a precipitator, so that the pH of the mixture could be easily adjusted. The deposition was done in electrolyte with Ga_2_O_3_ molarity of 1.0 M (pH 6) at a constant current density of 0.15 A/cm^2^. The Si substrate was cleaned with modified RCA cleaning using ethanol, acetone and DI water prior to the deposition in order to remove a native oxide layer. The growth time was fixed at 2 h. In this electrochemical process, a platinum (Pt) wire was used as an anode and Si substrate as a cathode. The schematic of electrochemical setup is shown in Figure 1a. After the deposition, the sample was immersed into the DI water to remove any unwanted residue.

The electrochemically deposited Ga_2_O_3_ was ammoniated in a quartz tube furnace as shown in Figure 1b, at various times of 15, 30 and 45 min and temperatures of 800°C, 850°C and 900°C under a flow of ammonia (NH_3_) gas of 100 sccm at atmospheric pressure. The timing chart of ammoniating process is shown in Figure 1c. Before starting the ammoniating process, the sample was put inside the quartz tube furnace and then the nitrogen (N_2_) gas was purged for 10 min to flush out the air in the quartz tube. After that, the temperature was increased up to the setting temperatures, i.e. 800°C, 850°C and 900°C from room temperature (RT) with a ramping rate of 28°C/min. After reaching the setting temperature, N_2_ gas was stopped and NH_3_ gas was introduced into the furnace. The furnace was immediately switched off upon reaching the setting ammoniating time. At the same time, NH_3_ gas was immediately stopped and N_2_ gas was purged back into the furnace for 1 h to remove the remaining NH_3_ gas during the cooling down process. The ammoniated structures were characterized using field-emission scanning electron microscopy (FESEM; Hitachi SU8083, Hitachi, Ltd, Chiyoda-ku, Japan), energy dispersive X-ray (EDX) spectroscopy and X-ray diffraction (XRD; Bruker D8 Advance, Bruker AXS, Inc., Yokohama-shi, Japan).

## Results and discussion

The nitridation of Ga_2_O_3_ to form GaN can be described as follows. Firstly, NH_3_ decomposes to N_2_ and H_2_ at high temperatures as illustrated by Equation 1 [29]. Then, Ga_2_O_3_ structures are deoxidized by H_2_ to form gaseous Ga_2_O as illustrated by Equation 2. Finally, GaN structure is synthesized through the reaction of Ga_2_O and NH_3_.

(1) $$2NH_{3\;}\left(g\right)\to N_{2}\left(g\right)+3H_{2}\;\left(g\right)$$

(2) $${\mathrm{Ga}}_{2}O_{3}\left(s\right)+\;2H_{2}\left(g\right)\to {\mathrm{Ga}}_{2}O\left(g\right)+2H_{2}O\left(g\right)$$

(3) $$Ga_{2}O\left(g\right)+2NH_{3}\left(g\right)\to 2GaN\left(s\right)+2H_{2}\left(g\right)+H_{2}O\left(g\right)$$

**Figure 2:**
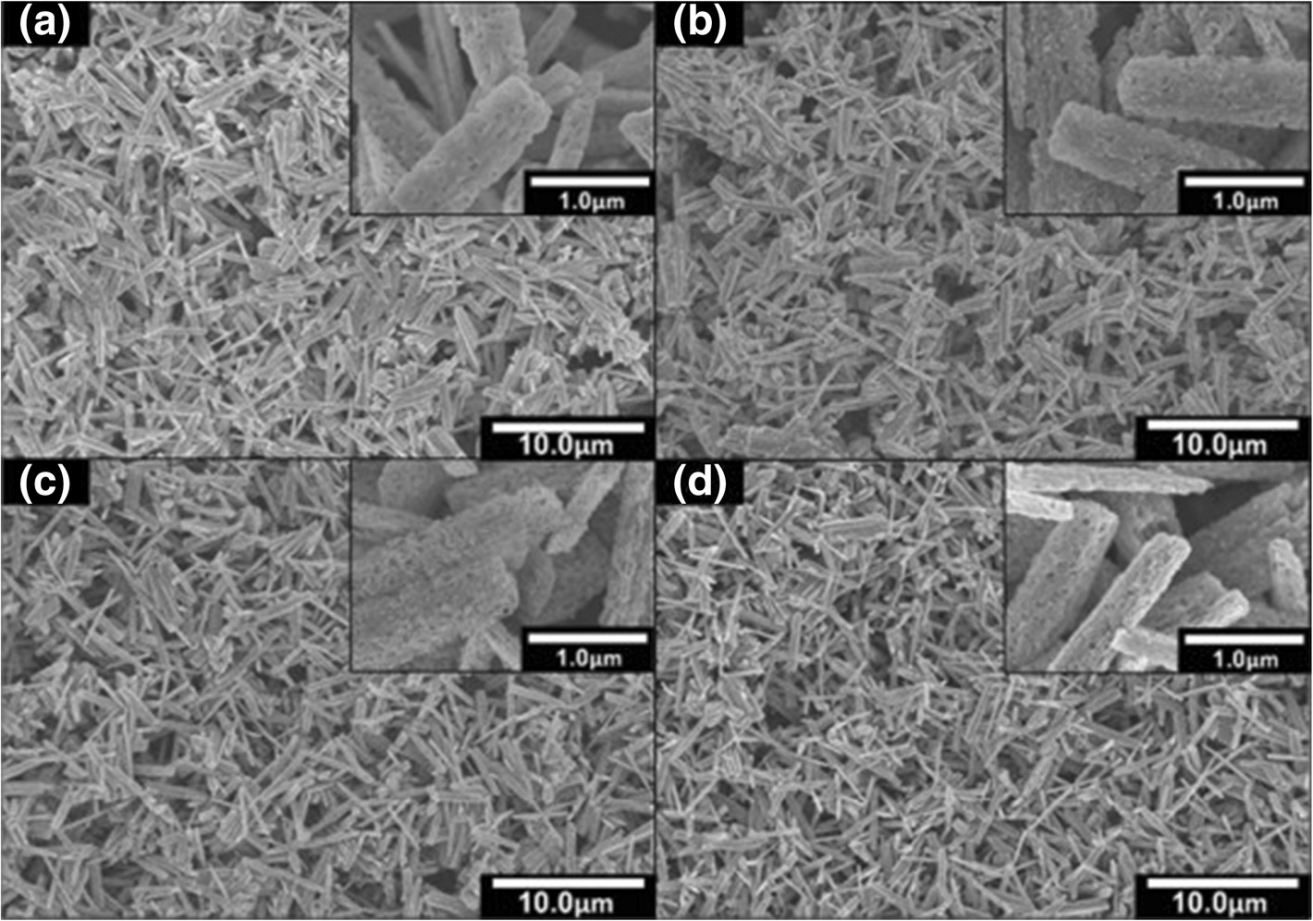
**Top view FESEM images of samples. (a)** Before nitridation, **(b)** after 15 min, **(c)** after 30 min and **(d)** after 45 min of nitridation at 850°C

**Figure 3:**
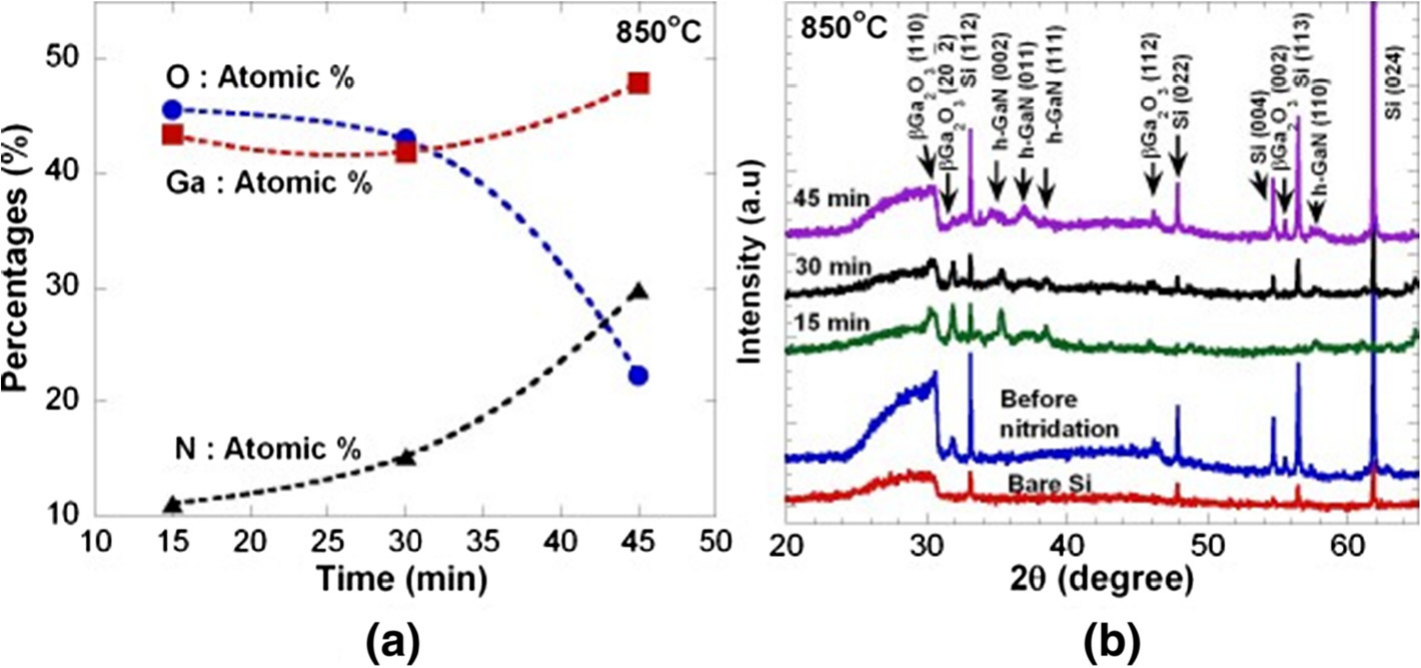
**EDX analysis and XRD spectra. (a)** Atomic percentage obtained from the EDX analysis and **(b)** XRD spectra of the samples nitridated at 850°C with various nitridation times.

Figure 2a shows the top view FESEM images of the electrochemically deposited Ga_2_O_3_ nanorods on Si substrate before the ammoniating process was applied. The lengths and diameters of the grown Ga_2_O_3_ nanorods were estimated to be in the range of 1,000 to 4,200 nm and 200 to 1,000 nm, respectively. It is noted that the measured EDX spectra confirm the elements of Ga and O, indicating the formation of Ga_2_O_3_ nanostructure. Figure 2b,c,d shows the top view FESEM images after the ammoniating process at 850°C for 15, 30 and 45 min, respectively. In a glance, it can be seen that there is no significant change of morphology taking place even at long ammoniating time of 45 min. However, it can be seen that there is a slight change on the surface of nanorods where the numbers of ‘hole’ increase with the ammoniating time. From the EDX analysis as shown in Figure 3a, the atomic percentage of N increases with ammoniating time while the atomic percentage of O decreases and the drastic changes seem to take place after ammoniating time of 30 min. Figure 3b shows the XRD spectra of samples before and after the ammoniating process at 850°C for 15, 30 and 45 min together with the XRD spectra of bare Si. In the bare Si, five peaks were observed at 33.1°, 47.8°, 54.7°, 56.4° and 61.8° corresponding to Si(112), Si(002), Si(004), Si(113) and Si(024), respectively. The as-grown Ga_2_O_3_ nanorods show three prominent peaks at 31.8°, 46.2° and 55.5° corresponding to β-Ga_2_O_3_(202), β-Ga_2_O_3_(112) and β-Ga_2_O_3_(002), respectively. After being ammoniated for 15, 30 and 45 min, several peaks corresponding to hexagonal GaN (h-GaN) planes were observed. However, β-Ga_2_O_3_-related peaks were still detected in all grown samples suggesting that β-Ga_2_O_3_ structures are not completely being transformed to GaN. From these XRD results, it can be said that an ammoniating time is not a dominant parameter since the complete transformation of Ga_2_O_3_ to GaN does not occur even at a long ammoniating time of 45 min.

**Figure 4:**
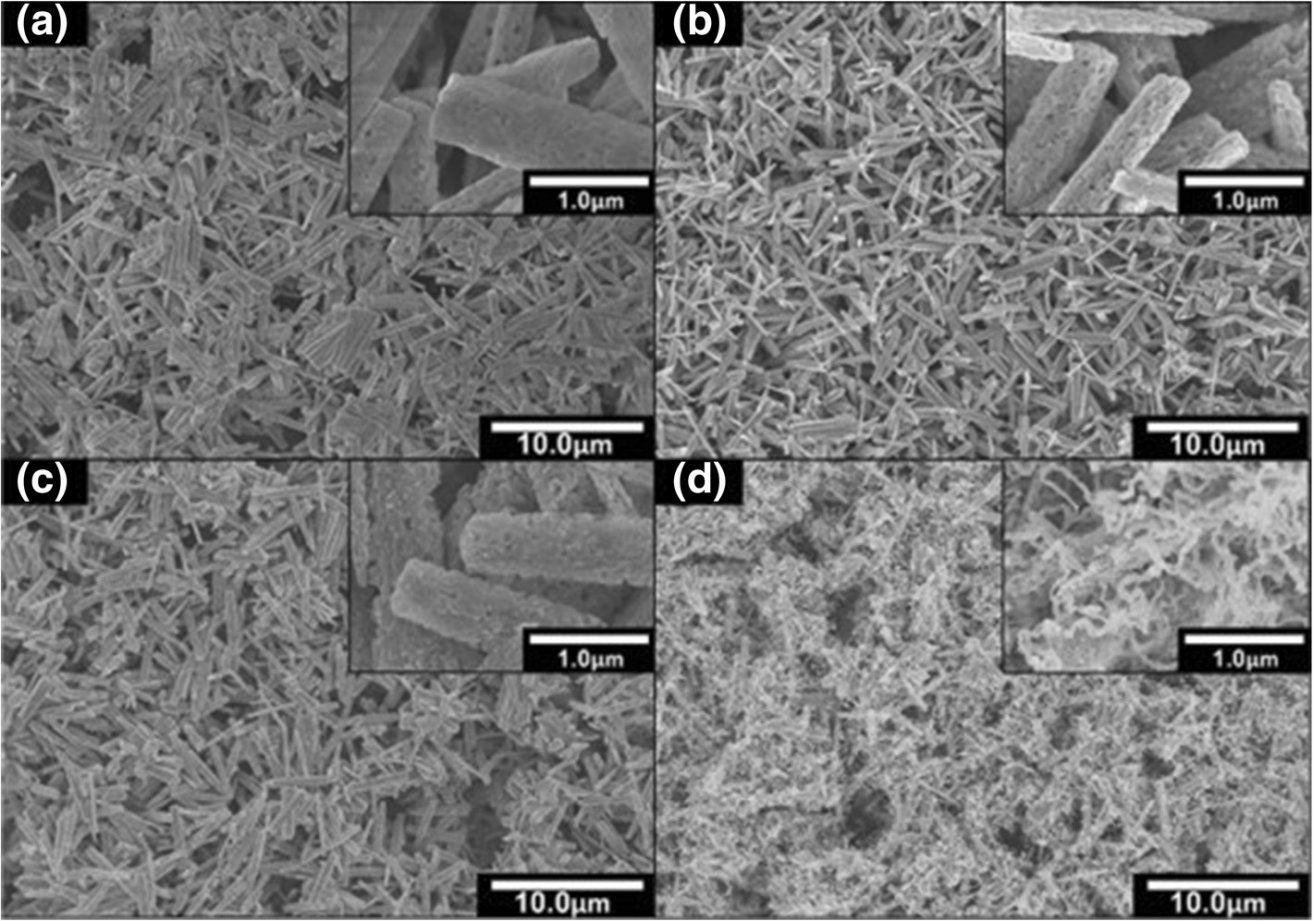
**Top view FESEM images of samples. (a)** Before nitridation, **(b)** after nitridation at 800°C, **(c)** after nitridation at 850°C and **(d)** after nitridation at 900°C for 15 min.

**Figure 5:**
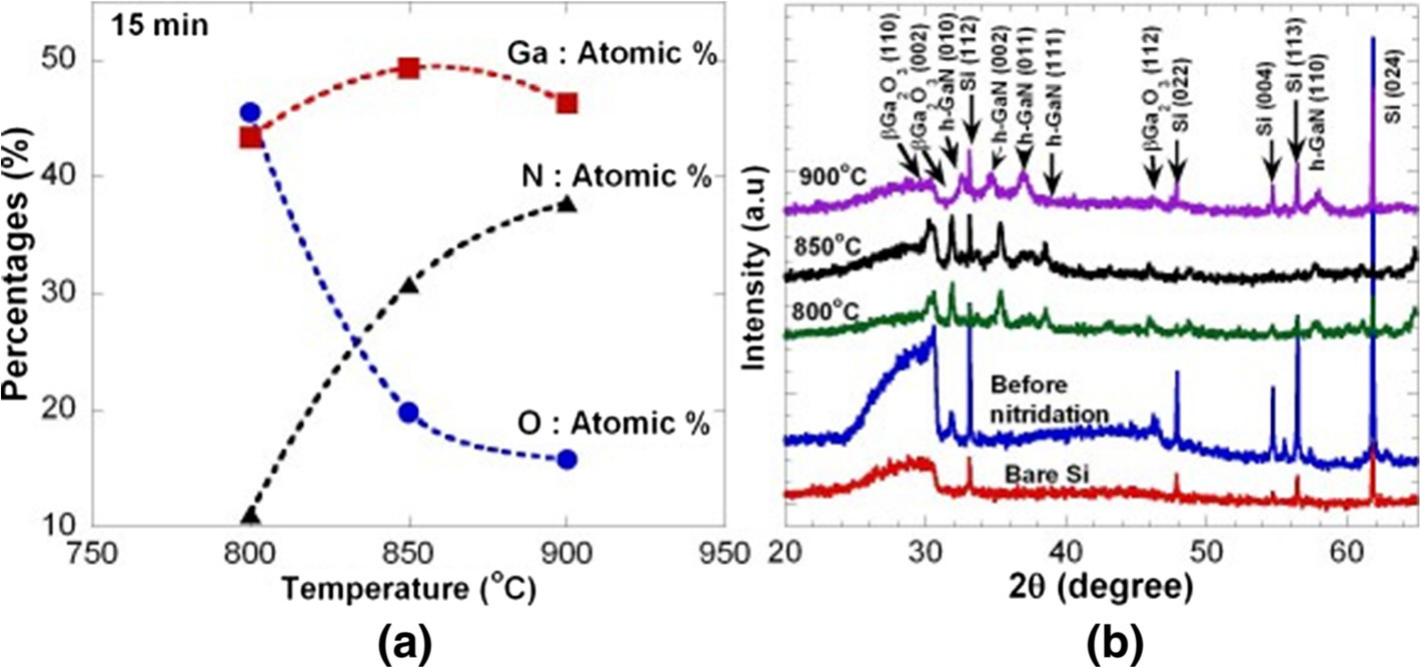
**EDX analysis and XRD spectra. (a)** Atomic percentage obtained from the EDX analysis and **(b)** XRD spectra of the samples nitridated at various temperatures for 15 min.

Figure 4a,b,c,d shows the FESEM images for the as-grown sample and the ammoniated samples at temperatures of 800°C, 850°C and 900°C for 15 min, respectively. It is noted here that β-Ga_2_O_3_ nanorods as shown in Figure 4a were also grown using the similar procedures and conditions with the samples shown in Figure 2 where their properties are similar. After being ammoniated at 800°C and 850°C, the general shape of the nanorods is still being preserved where no significant change in their morphologies is observed, as shown in Figure 4b,c, respectively. However, the same phenomenon with the samples shown in Figure 2 was observed where the numbers of ‘hole’ structures in the nanorods increase with the temperature. When the sample was further ammoniated at 900°C, as shown in Figure 4d, the structures have changed from nanorods to nanowires with the estimated lengths of 0.4 to 1.0 μm and diameters of 0.05 to 0.08 μm. This result is consistent with the result reported by Kim et al. where they claimed that the change of morphology could be significantly affected by the ammoniating temperature [30]. Figure 5a shows the EDX analysis for the samples ammoniated at temperatures of 800°C, 850°C and 900°C. It can be seen that the atomic percentage of N increases with temperature while the atomic percentage of O decreases. As shown in Figure 5a, the drastic change in the atomic percentage seems to take place from 800°C to 850°C. Figure 5b shows the XRD spectra of the as-grown samples and the ammoniated samples at temperatures of 800°C, 850°C and 900°C for 15 min together with the XRD spectra of bare Si. After being ammoniated at 800°C, 850°C and 900°C, several h-GaN-related peaks were observed. However, as expected, β-Ga_2_O_3_-related peaks are being detected for the samples ammoniated at 800°C and 850°C, suggesting the incomplete transformation of β-Ga_2_O_3_ to GaN. It can be simply said that the complete transformation was achieved at ammoniating temperature of 900°C where no β-Ga_2_O_3_-related peak was observed except h-GaN peaks. The peaks related to GaN structures were detected at 32.5°, 34.6°, 36.9° and 58.0° corresponding to h-GaN(010), h-GaN(002), h-GaN(011) and h-GaN(110), respectively. The obtained results show a similar tendency with the results reported by Li et al*.*[31] where the complete transformed GaN with hexagonal wurtzite structure was obtained at a temperature of 900°C. It is noted that from the EDX analysis, the atomic percentage of O is not zero in the sample of 900°C. It is speculated that the detected O element is contributed by a native oxide layer in the sample after being exposed to air ambient. From these XRD results, it can be said that an ammoniating temperature is the key parameter in producing the complete transformation of Ga_2_O_3_ to GaN since it takes only a short ammoniating time of 15 min.

**Figure 6:**
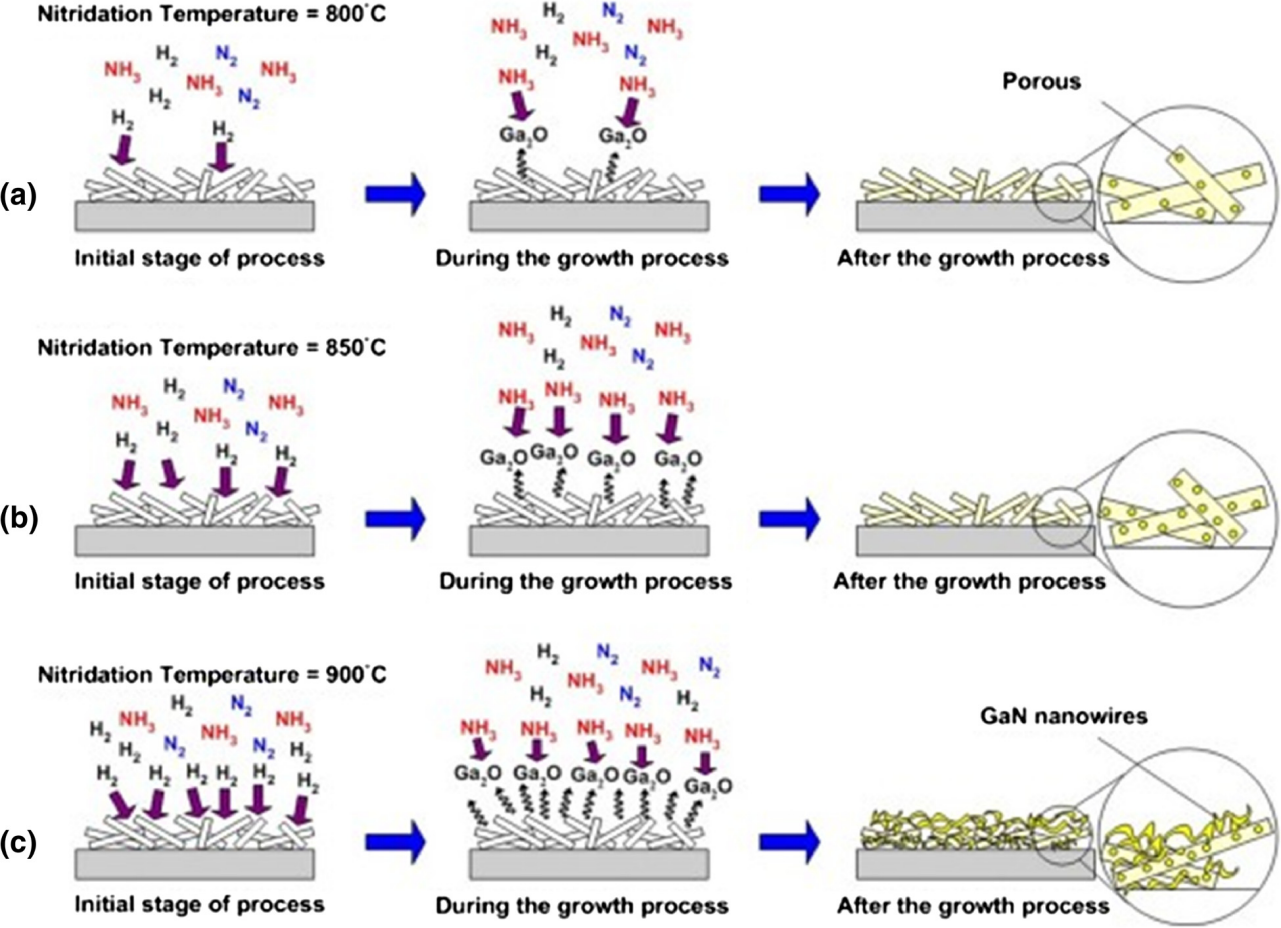
**Proposed growth mechanism for the transformation Ga**_**2**_**O**_**3 **_**to GaN.** At ammoniating temperatures of **(a)** 800°C, **(b)** 850°C and **(c)** 900°C.

Based on the obtained morphological and structural properties, the reaction mechanism for the transformation of Ga_2_O_3_ to GaN under a flow of ammonia with the dependence of temperatures is proposed and discussed. In principle, as shown by Equation 1, with the increase of temperature, the decomposition rate of NH_3_ should be increased, resulting to the generation of a high density of H_2_. Then, this leads to the higher deoxidization or vaporization rate of Ga_2_O_3_ structures to form gaseous Ga_2_O, as illustrated by Equation 2 [32]. Finally, this situation would increase the reaction of gaseous Ga_2_O and NH_3_ to promote the formation of a GaN structure. This means that the transformation of Ga_2_O_3_ to GaN does not proceed without the deoxidation or vaporization of Ga_2_O_3_ or, in other words, without the existence of gaseous Ga_2_O. Due to such deoxidization and reaction activities, it is speculated that the original morphological structure of Ga_2_O_3_ will be diminished and a new morphological structure of GaN will be formed. As shown in Figure 4b, the SEM image of the ammoniated structure at temperature of 800°C shows no significant change of the morphology as compared to the unammoniated structure shown in Figure 4a. This seems to show that the temperature of 800°C is considerably low for the aggressive deoxidization or vaporization of Ga_2_O_3_ to take place, resulting to low reaction of gaseous Ga_2_O and NH_3_ to form GaN. This also agrees with the XRD analysis as shown in Figure 5b where the sample ammoniated at temperature of 800°C contains a mixture of Ga_2_O_3_ and GaN structures. Figure 6a illustrates the growth mechanism for the temperature of 800°C. When the temperature is increased to 850°C, the morphology of the ammoniated structure shows a slight change where the numbers of ‘hole’ structures seem to be increased as shown in Figure 4c. It can be said that the deoxidization or vaporization rate of Ga_2_O_3_ slightly increases to form gaseous Ga_2_O. However, since the original structure of Ga_2_O_3_ does not diminish and the XRD spectra also shows a mixture of Ga_2_O_3_ and GaN, it can be said that the temperature of 850°C is still not favorable to drastically increase the density of gaseous Ga_2_O to continuously react with NH_3_ to form a GaN structure. Figure 6b illustrates the growth mechanism for the temperature of 850°C. At high temperature of 900°C, as shown in Figure 4d, it can be seen that the original nanorod structure of Ga_2_O_3_ has diminished and a new structure in the form of nanowire-like structure has been produced. It can be said that temperature of 900°C seems to be high enough to realize a complete deoxidization of Ga_2_O_3_ to generate a high density of gaseous Ga_2_O. Thus, the reaction of gaseous Ga_2_O and NH_3_ seems to be well promoted at such condition to form GaN nanowires. It is noteworthy that during the ammoniating process at temperature of 900°C, white ‘cloudy’ ambient was observed in the furnace which seems to indicate the aggressive deoxidization of Ga_2_O_3_ to form Ga_2_O gaseous. After ammoniating process, it was found that the original white colour of the as-grown Ga_2_O_3_ diminished and new structures in light yellow colour appeared. The XRD spectra as shown in Figure 5b reveals that the detected peaks belong only to the structures of GaN. It can be said that the temperature of 900°C is favorable to drastically increase the deoxidization rate of Ga_2_O_3_ to generate gaseous Ga_2_O to continuously react with NH_3_ to form GaN with new morphological nanostructures.

## Conclusions

The nitridation of the electrochemically grown Ga_2_O_3_ nanostructures to form GaN nanostructures on Si platform have been studied by varying the ammoniating times and temperatures. The complete transformation of Ga_2_O_3_ nanorods to h-GaN nanowires was achieved at 900°C with a short ammoniating time of 15 min. The obtained results suggest that the effect of the ammoniating temperature in realizing a complete transformation is more prominent than the ammoniating time. Form the proposed growth mechanism, it was found that a complete transformation to GaN nanostructures can not be realized without a complete deoxidization of Ga_2_O_3_. In a complete transformation process, it seems to show the occurence of morphological change. The presented method seems to be promising for the formation of h-GaN nanostructures on Si for the applications in sensing and optoelectronics.

## Competing interests

The authors declare that they have no competing interests.

## Authors’ contributions

NMG designed and performed the experiments, participated in the data analysis and prepared the manuscripts. KY participated in the nitridation process and revision of manuscript. AMH conceived the study, designed the experiments, participated in the data analysis and revised the manuscript. All authors read and approved the final manuscript.
